# Analytical and Finite Element Analysis of the Rolling Force for the Three-Roller Cylindrical Bending Process

**DOI:** 10.3390/ma17215230

**Published:** 2024-10-27

**Authors:** Doina Boazu, Ionel Gavrilescu, Felicia Stan

**Affiliations:** 1 Department of Mechanical Engineering, Dunarea de Jos University of Galati, Domneasca 47, 800008 Galati, Romania; doina.boazu@ugal.ro; 2 Independent Researcher, 800193 Galati, Romania; ionel.gavrilescu@gmail.com; 3 Center of Excellence Polymer Processing, Dunarea de Jos University of Galati, Domneasca 47, 800008 Galati, Romania

**Keywords:** three-roll bending process, finite element analysis, rolling force, pre-tensioning force, pipe steel

## Abstract

In the roll bending process, the rolling force acting on the roller shafts is one of the most important parameters since, on the one hand, it determines the process settings including the pre-loading, and, on the other hand, its distribution and size may affect the integrity of both the bending system and the final product. In this study, the three-roller bending process was modeled using a two-dimensional plane–strain finite element method, and the rolling force was determined as a function of plate thickness, upper roller diameter, and yield strength for various API steel grades. Based on the numerical simulation results, a critical bending angle of 41° was identified and the rolling systems were divided into two categories, of less than or equal to, and greater than 41°, and an analytical model for predicting the maximum rolling force was developed for each category. To determine the optimal pre-tensioning force, two optimization formulations were proposed by minimizing the maximum equivalent stress and the absolute maximum displacement. The rolling forces predicted by the analytical models were found to be in good agreement with the numerical simulation results, with relative errors generally less than 10%. The predictive analytical models developed in this study capture well the complex deformation behavior that occurs during the roll bending process of steel plates, providing guidelines and predictions for industrial applications of this process.

## 1. Introduction

In forming technology, roll-forming processes are used for manufacturing a wide variety of metal parts and semi-finished products, finding applications in various industries such as pressure vessels, steel structures, and shipbuilding. Among the roll-forming processes, the roll bending process (also called pipe rolling) is a manufacturing process that produces cylindrical and conical components (e.g., pipes, shells, barrels, and channels) by continuously bending a metal sheet between two or more symmetrical or un-symmetrical rollers [1,2]. The roll bending process is particularly suitable when large diameter, long pipes are needed; the semi-finished pipes being obtained by, most often, using symmetrical three-roller bending systems [3].

In order to remain competitive, one of the critical challenges faced by the plate- and sheet-bending industry is to engineer and deliver steel pipes with the wall thickness in a wider range of sizes for different types of steel grades using the existing roll bending machines. Therefore, the maximum force acting on the roller shafts during the rolling process must be accurately determined in order to maximize the bending capacity of the roll bending machines without overloading them and to calculate the required preloading [4,5,6,7]. However, the calculation of the rolling force acting on the roller shafts as a function of the material properties and process parameters, including yield stress, plate thickness, and constructive parameters of the rolling system among others, is still not straightforward, despite the technological interest.

A literature review on this subject indicates that several research papers have focused on the analytical and theoretical modeling of the roll bending process; among them, the works were mainly focused on the final radius of curvature of the pipe [8]; the position of the upper roller [9]; or the rolling force [5,6,10]. For instance, Gandhi and Raval [9] established analytical and empirical models to estimate the position of the top roller as a function of the final radius of curvature for the three-roller cylindrical-bending process. By equating the external bending moment and plate internal bending moment, Kim et al. [8] proposed an analytical model to predict the final radius of pipes manufactured using three-roller bending, accounting for various process and material parameters, and the results were compared with those obtained from numerical analysis. Yu et al. [7] developed the mechanism for a three-roller setting round process and showed that, in order to manufacture pipes of high precision, the tonnage of the rolling device must be greatly increased. Chudasama and Raval [5] considered various geometrical parameters and material properties and proposed an analytical model for the calculation of the bending force for the three-roller conical-bending process. Within this formulation, the shear forces were neglected while deriving the internal bending moment under plane–strain bending. Shinkin [6] investigated the rolling process using an asymmetric three-roller sheet-bending system and proposed a mathematical formulation for the reaction forces of the roller supports, including the extension of plastic deformation over the sheet thickness and the relative deformation of the longitudinal surface of the rolled sheet, considering different geometrical and material properties. Salem et al. [10] studied the manufacturing of cylindrical and conical sections using an asymmetrical three-roll bending process and developed an analytical model to predict the roll bending force, the residual stresses, and the power of the roll bending process that were verified against experimental data. However, with the reported analytical models, it is difficult to accurately predict the rolling force as a function of plate thickness and yield strength as they do not fully account for the plastic deformation. Thus, efforts are still being made to improve the predictive capability of the analytical models.

Due to the development of numerical methods along with commercial simulation software, finite element analysis (FEA) has become a common tool for product design and structure analysis, providing useful insights into complex problems such as metal forming processes [11,12]. Therefore, research efforts have been devoted to modeling and simulation of the roll-bending processes through the use of finite element methods, especially to validate the analytical models [13,14,15,16,17,18,19,20,21]. For instance, Ktari et al. [14] studied the three-roller bending process through 2D FE analysis, including the spring-back phenomenon, and experimental design, and generated maps for the curvature radii as a function of the distance between the two lower rollers and the position of the upper roller. Fu et al. [16] investigated the three-roll bending process using FEA in Abaqus/Explicit and Standard solvers and derived a relationship between the downward inner roller displacement and the desired spring-back radius (unloaded curvature radius) of the bent plate by both analytical and FE approaches, which agree reasonably well with the experimental results. Mohit and Gajare [18] studied the three-roller bending process using 3D dynamic FEA in Ansys and the simulation results, in terms of the maximum vertical displacement of the upper roller, were compared with experimental results. Jadhav and Talmale [19] carried out both analytical and FE analyses using Catia V5 and Ansys, concluding that the stress and strain values predicted with the analytical model are in good agreement with the FE simulation. Wang et al. [20] reported a theoretical model for the four-roller bending process based on the rebound theory of thick plates, which was compared with FE results based on the Abaqus/Explicit model. More recently, Gavrilescu et al. [21] proposed a hybrid numerical–analytical approach for the three-roller bending process, in which the plastic hinge condition observed during the FE analysis was coupled with the bent bar theory and two analytical models for estimating the bending force were derived. Furthermore, using geometric and deformation compatibilities, analytical expressions for the vertical displacement of the upper roller as a function of the curvature of the bending plate were also developed.

Although FE analysis can provide valuable information that cannot be obtained through experiments or may not be available any other way, the successful use of FE analysis as a standard tool still requires advanced understanding of FE methods in order to obtain reliable and accurate predictions. On the other hand, given the circumstances and the manufacturing capabilities, FEA is not always available or it is time consuming. Thus, analytical models are still preferred by the industry to estimate the rolling forces. However, as indicated by the literature review, analytical formulas do not adequately describe the material behavior, including plastic deformation in bending, thus under- or overestimating the rolling forces. Therefore, the objective of this study is to construct an analytical model for the rolling force by which the maximum rolling forces can be accurately estimated as a function of plate thickness and yield strength, exploiting the findings from the FE modeling of the rolling process. In addition, two optimization formulations are proposed by minimizing the maximum equivalent stress or the absolute maximum displacement, respectively, to determine the optimal pre-tensioning force.

## 2. Finite Element Simulation of the Three-Roller Bending Process

### 2.1. Research Methodology

**Figure 1:**
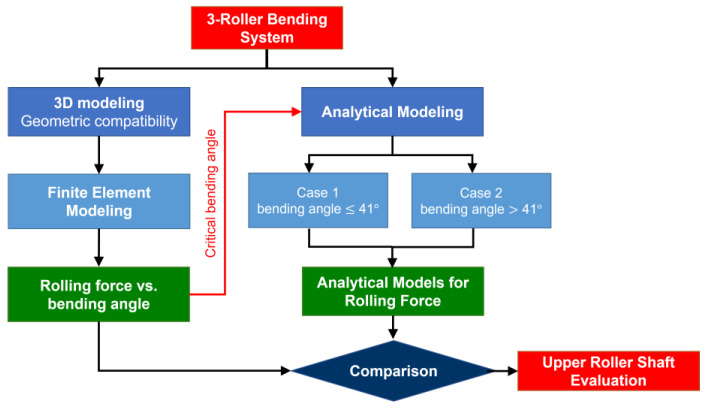
Research methodology for the determination of the rolling force.

Figure 1 shows the research methodology employed in this study for the determination of the rolling force. The methodology is divided into two steps. First, the rolling force was calculated using FEA, taking into account the geometric compatibility [21]. In the second step, the variation in the simulated rolling force as a function of the bending angle was analyzed and the sensitivity of the maximum rolling force as a function of the bending angle led to the observation of a critical bending angle. Therefore, the bending systems are divided, depending on the bending angle, into two categories (less than or equal to and greater than 41°), and an analytical formula to predict the rolling force as a function of plate thickness and yield strength is derived for each category. Furthermore, the upper roller shaft is verified using FEA and the optimal value for the pre-tensioning force is determined by minimizing the maximum equivalent stress or the absolute maximum displacement that will be explained in detail in Section 4.

### 2.2. Vertical Displacement of the Lower Rollers

**Figure 2:**
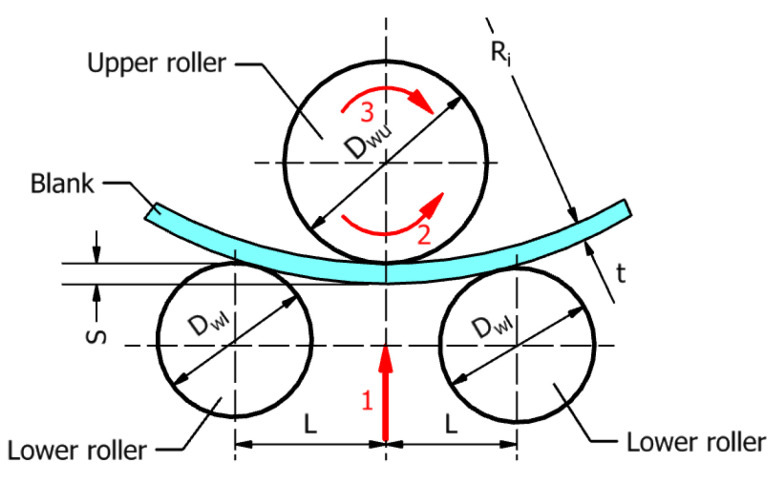
The basic principle of three-roller bending process and the main working phases.

The three-roller bending system comprises two lower roller shafts with diameter $D_{wl}$ and one upper roller shaft with diameter $D_{wu}$. In general, the operation of the three-roller bending system consists of three main working stages, as indicated in Figure 2.

(1) Vertical displacement of the lower roller assembly (the two lower rollers rotate freely around their own axes of rotation) while the upper roller is fixed. The vertical displacement of the lower rollers or tightening is denoted by *S*, in Figure 2;
(2) Rotation of the upper roller in one direction while the vertical position of the lower rollers is fixed;
(3) Rotation of the upper roller in the opposite direction to the second stage while the vertical position of the lower rollers is fixed.

**Figure 3:**
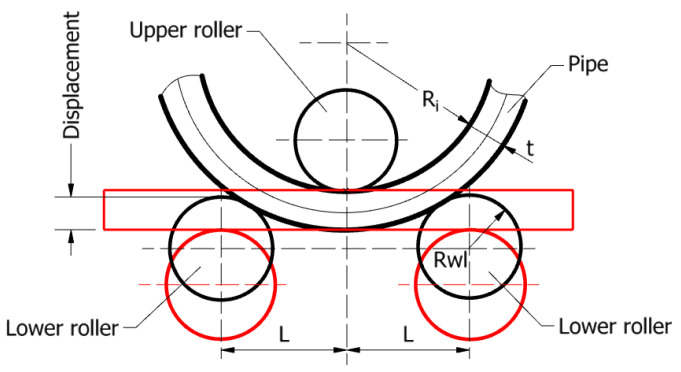
Schematic representation of the three-roller bending process with the vertical displacement of the lower rollers in geometric compatibility.

The vertical displacement of the lower rollers can be determined considering the geometric compatibility of the elements that come into contact (i.e., the outer surfaces of the three rollers and the surfaces of the plate), as shown in Figure 3, and corresponds to the determination of the maximum rolling force [21].

According to the geometric compatibility principle, the maximum vertical displacement of the assembly formed by the two lower rollers can be calculated by the following [21]:

(1) $$Displacement=R_{wl}-(\sqrt{{\left(R_{e}+R_{wl}\right)}^{2}-{\left(L\right)}^{2}}-R_{e}),$$

in which $R_{wl}$ is the radius of the lower roller, $R_{i}$ is the inner radius of the semi-finished pipe, *t* is the wall thickness of the pipe (thickness of the rolled plate), *L* is the distance between the axis of the lower rollers and the axis of the upper roller, and $R_{e}$ is the outer radius of the semi-finished pipe, $R_{e}=R_{i}+t$.

### 2.3. Two-Dimensional Modeling of the Three-Roller Bending Process

The steel grades used in the offshore oil and gas industry vary from Grade B to Grade X100 [22]. Therefore, in order to extend the three-roller bending system capabilities for different industrial sectors and engineering applications and to investigate the influence of steel strength on the rolling force, in this study, various API steels such as X70, X65, X60, X52, X42, and B were considered. The material properties used in the finite element simulation are given in Table 1 and were adopted from [22]. The steel plates were considered to have a nonlinear behavior (i.e., bilinear elastic–plastic behavior), whereas the elements of the rolling system (i.e., the upper and lower rollers) have a linear elastic behavior.

The geometrical parameters of the bending system are presented in Table 2. It should be noted that an industrial three-roller bending machine was considered, which can be equipped with a wide selection of upper roller shafts (Table 2), while the distance between the axis of the lower and upper rollers, *L*, is fixed to 400 mm.

In the simulation, the blank is a rectangular plate and its thickness varies from 14.3 up to 28.58 mm depending on the steel grade and applications. The blank length corresponds to the maximum length of the rolling machine, namely 1200 mm.

**Figure 4:**
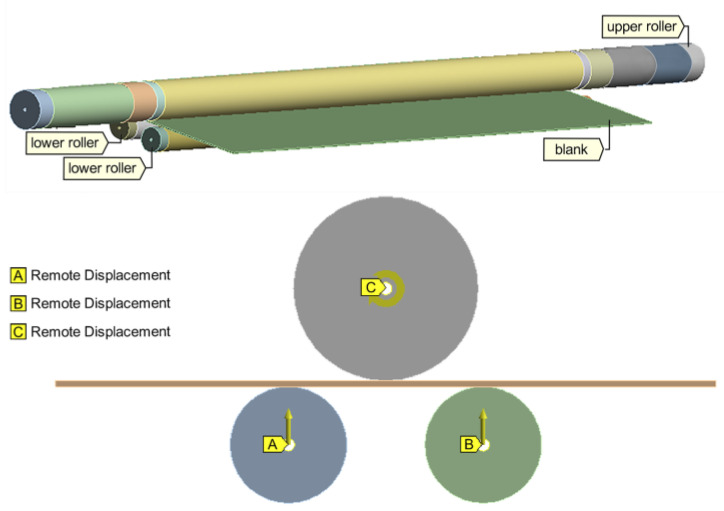
Elements of the three-roller bending system and the 2D FE model with applied boundary conditions.

The finite element analysis was carried out with the aim to determine the maximum rolling force. Therefore, two-dimensional (2D) plane–strain models were developed in ANSYS Workbench Static Structural analysis software (version 19.0, 2019, Ansys, Inc., Canonsburg, PA, USA [23]), as illustrated in Figure 4.

In the FE analysis, the three-roller bending process was abstracted into three main stages, as shown in Figure 2, and the boundary conditions were correlated with the rolling stages, as follows:

(i) Stage 1—the lower rollers raise with a vertical displacement determined by geometric compatibility (roller tightening);
(ii) Stage 2—the lower rollers have fixed axes (the rotation around the axes is free) and the upper roller has an imposed rotation;
(iii) Stage 3—the lower rollers have fixed axes (the rotation around the axes is free) and the upper roller has an imposed rotation in the opposite direction to Stage 2.
(iv) It should be noted that the axis of the upper roller shaft is fixed in all three stages.

**Figure 5:**
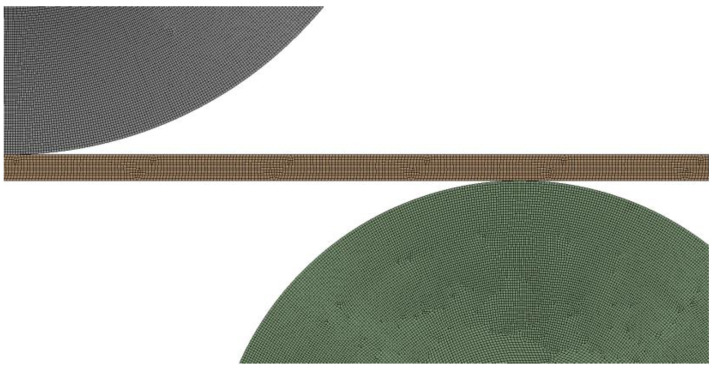
Finite element model of the three-roller bending system—contact zone between the steel plate and the upper and lower rollers.

Figure 5 shows a typical finite element mesh for the rolling process. The plate was modeled with the element type PLANE 183, which is a 2D eight-node quadrilateral higher order element with plasticity, large deflection, and large strain capabilities [23]. The element size was set at approximately 2 mm. The frictional contact conditions are identical for all three rollers. The contact between the rollers and the sheet is accounted for using a friction coefficient equal to 0.6, which corresponds to dry friction.

FE analysis was performed by varying the yield strength, the diameter of the upper roller, and the plate thickness, and the reaction force was determined from the axis of the upper roller. The reaction forces were uniformly distributed per unit length, thus the unit is N/m. It should be noted that maximum rolling force was reached at the end of Stage 1, and corresponds to the vertical component of the reaction force.

## 3. Analytical Modeling of the Rolling Force

During the roll bending process, very high forces are developed that can affect the normal operation and integrity of the rolling machines, especially if a small spacing between the lower rollers is employed. Therefore, the force acting on the upper roller shaft must be reduced to suppress the deflection in the central area, particularly in the case of rolling thick-walled long pipes.

In order to reduce the rolling forces, three different constructive solutions were identified in the literature [24], as follows:

(1) The spacing of the two lower roller shafts, 2*L*, and the tightening, *S*, of the upper roller with respect to the lower rollers satisfy the conditions:
  (2) $$2L>D_{wu}+D_{wl}\;\mathrm{and}\;S>D_{wl}/2,$$
  where $D_{wu}$ is the diameter of the upper roller, and $D_{wl}$ is the diameter of lower rollers, as illustrated in Figure 2;
(2) The spacing of the lower rollers, 2*L*, satisfies the condition:
  (3) $$OD+D_{wl}>2L\geq 0.85(OD+D_{wl}),$$
  where OD is the outer diameter of the pipe (bended plate);
(3) Pre-bending the ends of the steel plate beforehand in a press or a roller mill.

In practice, in order to reduce the bending deformations produced by the rolling forces in the central area, the upper roller is pre-tensioned (pre-stressed) by applying forces on its ends, generating a vertical deformation in the opposite direction to that produced by the rolling forces.

In the present study, in order to develop an analytical model for predicting the rolling force, the findings on the bending angle from the FE analysis were taken into account and correlated with the observations regarding the spacing between the lower rollers [24]. After performing a significant number of numerical simulations (see Session 2.2), taking into account different combinations of steel grade, plate thickness, upper and lower roller diameters and spacing between the lower rollers, the variation in the simulated rolling force as a function of the bending angle was analyzed and the sensitivity of the maximum rolling force as a function of the bending angle led to the observation of a critical bending angle. Therefore, two cases were identified, depending on the bending angle, as follows:

(i) Case 1: Rolling systems with a bending angle less than or equal to 41°, in which case, the ratio between the diameter of the upper and lower shaft rollers is greater than 1.3.
(ii) Case 2: Rolling systems with a bending angle greater than 41°, in which case, the ratio between the diameter of the upper and lower rollers is less than 1.2.

Note, however, that in both cases, permanent (plastic) deformation in the outer and inner fibers of the plate must be achieved, and the following relationship must apply [2]:

(4) $$\frac{D_{i}}{t}<\frac{E}{\sigma_{Y}}+1,$$

in which $D_{i}$ is the inner diameter of the rolled plate, *t* is the thickness of the plate, *E* is the Young’s modulus, and $\sigma_{Y}$ is the yield stress of the plate.

### 3.1. Roll Bending Systems with a Bending Angle Less than or Equal to 41°

**Figure 6:**
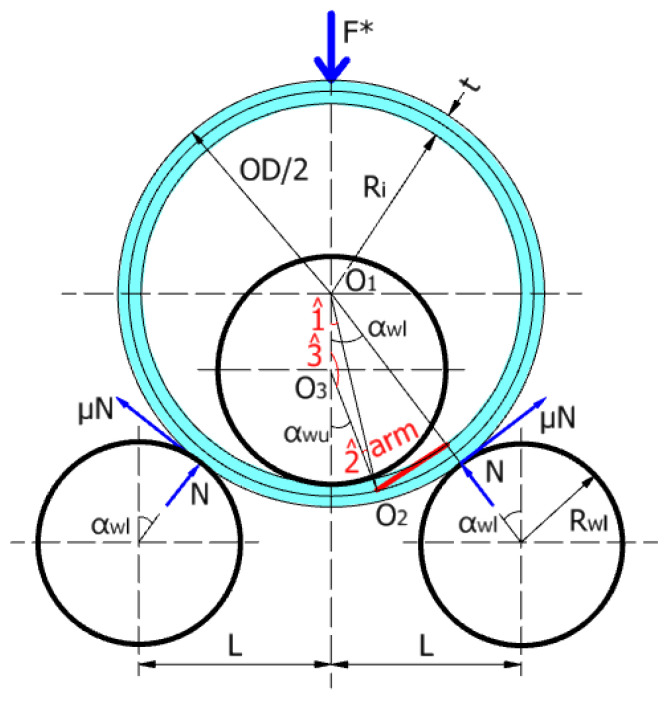
Schematic representation of the rolling system with $\alpha_{wl}\leq 41{}^{\circ}$.

Figure 6 presents the schematic representation of the three-roller bending system with a bending angle less or equal to 41°. For this particular case, the distribution of normal stress along the cross-section, in the tangent area of the upper roller, corresponds to the so-called plastic hinge [21].

The assumptions used to calculate the bending force in this particular case are as follows: (i) the coefficient of friction between the rollers and plate is 0.6, and (ii) the bending moment in the cross-section of the plate, in the tangent area of the upper roller (i.e., the section with the center in O_2_), corresponds to the complete plastified section of the plate [21]. Thus, for plastic bending, the bending moment M can be calculated by [2,21].

(5) $$M=K_{1}\times \sigma_{Y}\times \frac{b\times t^{2}}{4},$$

where *b* is the width of plate, *t* is the thickness of the plate (i.e., the wall thickness of the rolled pipe), $\sigma_{Y}$ is the material yield limit, and $K_{1}$ is a reinforcement coefficient that depends on the ratio $\frac{t}{R_{i}}$ [25].

The $K_{1}$ coefficient varies in the range of 1.1–1.25 [25]. Since no specific details on how to choose the value of the reinforcement coefficient $K_{1}$ are given in [25], the dependence of $K_{1}$ on the $t/R_{i}$ ratio was determined based on the FE simulations, and are presented in Table 3.

The bending angle $\alpha_{wl}$ can be expressed as

(6) $$\hat{\alpha_{wi}}=arcsin(\frac{L}{\frac{OD}{2}+R_{wl}}),$$

where OD is the outer diameter of the pipe, $R_{wl}$ is the radius of lower roller shaft, and L is the half of the spacing between the lower rollers.

From the notations given in Figure 6, it follows that

(7) $$R_{wu}\times \hat{\alpha_{wu}}=R_{wl}\times \hat{\alpha_{wl}}.$$

Based on Equations (6) and (7), after simplification, the winding angle can be computed by

(8) $$\hat{\alpha_{wu}}=\frac{R_{wl}\times \hat{\alpha_{wl}}}{R_{wu}}$$

Using the sine theorem, from the triangle O_1_O_2_O_3_ in Figure 6, the following relationships can be defined:

(9) $$\frac{R_{i}-R_{wu}}{sin(\hat{2})}=\frac{R_{wu}+\frac{t}{2}}{sin(\hat{1})}=\frac{R_{i}+\frac{t}{2}}{sin(180-\hat{\alpha_{wu}})}$$

(10) $$\frac{R_{wu}+\frac{t}{2}}{sin(\hat{1})}=\frac{R_{i}+\frac{t}{2}}{sin(\hat{\alpha_{wu}})}$$

(11) $$\hat{1}=arcsin\left[\left(R_{wu}+\frac{t}{2}\right)\times sin(\hat{\alpha_{wu}})\times \frac{1}{R_{i}+\frac{t}{2}}\right]$$

where $R_{i}$ is the inner radius of the rolled plate, *t* is the thickness of the plate, $R_{wl}$ is the radius of the lower roller shaft, $R_{wu}$ is the radius of the upper roller shaft, and $\alpha_{wu}$ is the winding angle on the upper shaft.

Then, the vertical reaction force and the rolling force (the pressure force) are given by the following:

(12) $$F=N\times \cos\left(\hat{\alpha_{wl}}\right)+\mu \times N\times \sin\left(\hat{\alpha_{wl}}\right)$$

(13) $$F^{*}=2\times F$$

in which, the normal force can be computed by

(14) $$N=\frac{M}{arm}$$

with

(15) $$arm=O_{1}O_{2}\times sin\left(\hat{\alpha_{wl}}-\hat{1}\right)={(R}_{i}+\frac{t}{2})\times sin\left(\hat{\alpha_{wl}}-\hat{1}\right).$$

### 3.2. Roll Bending Systems with a Bending Angle Greater than 41°

For this particular bending case, the formulation for the rolling force had a starting point in the work proposed by the Vukota [2] and Patrick et al. [3].

According to Vukota [2], the bending moment in the purely plastic domain for a rectangular cross-section is given by

(16) $$M=n\times (UTS)\times \frac{b\times t^{2}}{4}$$

where *n* is a correction coefficient that accounts for the material hardening (*n* = 1.6 to 1.8 [2]), *UTS* is the ultimate tensile strength of the material, *b* is the width of beam (length of bending), and *t* is the plate thickness.

Another expression for the calculation of the bending moment in the purely plastic domain was proposed by Patrick et al. [3] as follows:

(17) $${M=K}_{1}{\times \sigma }_{Y}\times \frac{b\times t^{2}}{4}$$

where $K_{1}$ is a reinforcement coefficient, $\sigma_{Y}$ is the material yield limit, and *b* and *t* are the width and the thickness of the plate, respectively.

Starting from the expressions for the bending moments (16) and (17), taking into account the observations from the FE analysis for the bending angle $\alpha_{wl}>41{}^{\circ}$, in this study, a new formulation for the bending moment in the purely plastic domain is proposed, which is further used to obtain the expression for the rolling force.

**Figure 7:**
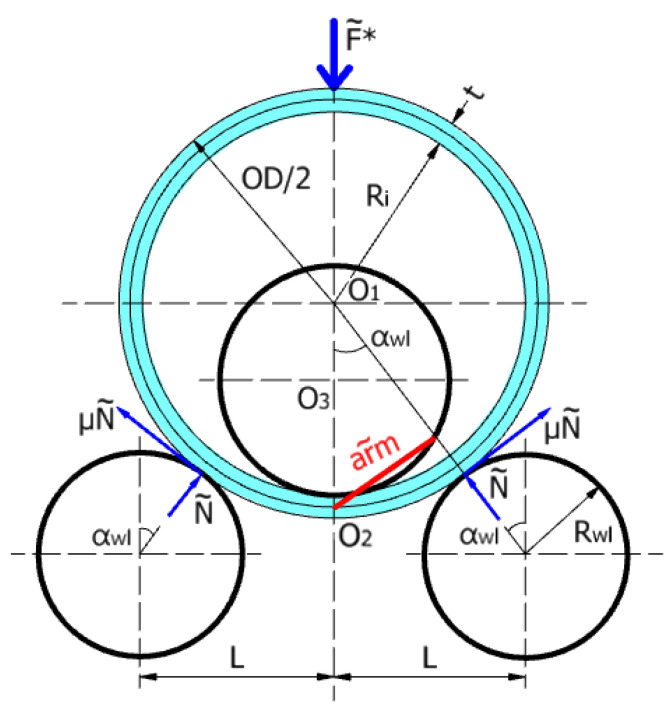
Rolling system with a bending angle $\alpha_{wl}>41{}^{\circ}$.

In order to calculate the rolling force in the case of a three-roller bending system with a bending angle $\alpha_{wl}>41{}^{\circ}$, the following assumptions are introduced: (i) the coefficient of friction between the rollers and the steel plate is 0.6, and (ii) the bending moment of the cylindrical section is the moment in the O_2_ center of the sheet in the axis of symmetry of the system (Figure 7).

The bending moment corresponding to the bending moment in the purely plastic domain for a rectangular cross-section, in the axis of symmetry of the system, is given by the following:

(18) $$\tilde{M}=K_{1}{\times K_{2}\times \sigma }_{Y}\times \frac{b\times t^{2}}{4}$$

where *b* is the width of plate (i.e., one meter), *t* is the thickness of the rolled plate, $\sigma_{Y}$ is the material yield limit, $K_{1}$ is the reinforcement coefficient that depends on the ratio $\frac{t}{R_{i}}$ (as given in Table 3), and $K_{2}$ is a coefficient that is defined by the following:

(19) $$K_{2}=\frac{\left(OD-2\times t\right)}{{2\times R}_{wu}}$$

in which OD and *t* are the outer diameter and the thickness of the rolled plate, respectively, and $R_{wu}$ is the radius of the upper roller shaft.

It should be noted that, within the proposed formulation, a new coefficient $K_{2}$ was introduced that takes into account the ratio between the inner radius of the rolled plate (pipe) and the radius of the upper roller, $R_{i}/R_{wu}$, as can be seen in Equation (19).

The normal and vertical reactions and the rolling force are given by the following relationships:

(20) $$\tilde{N}=\frac{\tilde{M}}{\tilde{arm}}$$

(21) $$\tilde{F}=\tilde{N}\times \cos\left(\hat{\alpha_{wl}}\right)+\mu \times \tilde{N}\times sin(\hat{\alpha_{wl}})$$

(22) $${\tilde{F}}^{*}=2\times \tilde{F}$$

in which the arm and the bending angle $\alpha_{wl}$ can be calculated as follows:

(23) $$\tilde{arm}=\frac{1}{2}\times \left(OD-t\right)\times sin\left(\hat{\alpha_{wl}}\right)$$

(24) $$\hat{\alpha_{wl}}=arcsin(\frac{L}{\frac{OD}{2}+R_{wl}})$$

where OD is the outer diameter of the pipe, $R_{wl}$ is the radius of lower roller, and *L* is the half of spacing between the lower rollers.

## 4. Evaluation of the Upper Roller Shaft

In practice, there are many situations in which the metal forming industry must expand the production capabilities or maximize the bending capacity by introducing new ranges of materials or sheet thicknesses. Therefore, special attention must be given to the pre-tensioning forces and the level of stresses and vertical displacement of the upper roller shaft, especially when long length upper roller shafts are used (for instance, the lengths of pipes could be in the range of 9 to 12 m). In order to reduce the maximum vertical displacements in the working area, pre-tensioning systems on the upper shaft ends are required while the semi-finished product is perfectly centered with respect to the symmetry plane of the active area. Therefore, it is necessary to accurately estimate the pre-tensioning forces applied on the ends of the upper roller shaft, without overloading it. It should be noted that, in practice, the assembly of the lower rollers has additional stiffening using another two pairs of rollers.

**Figure 8:**
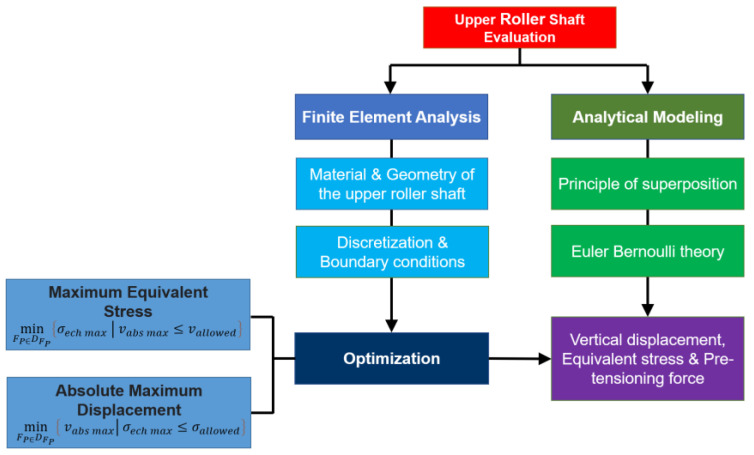
Flow chart for the verification of the upper roller shaft.

Figure 8 shows the methodology used in this study to determine the maximum equivalent stress and maximum vertical displacement of the upper roller shaft as well as the pre-tensioning force. The analytical approach is based on the Euler–Bernoulli theory, while for the FE analysis, the methodology involves two optimization procedures.

### 4.1. Calculation of the Maximum Stress and Displacement of the Upper Roller Shaft Using the Beam Theory

**Figure 9:**
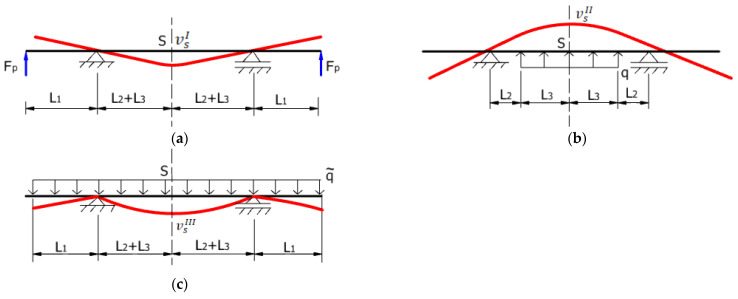
Schematic representation of the three individual loading stages: (**a**) load from pre-tensioning forces; (**b**) load from roll bending; (**c**) load from the weight of the upper roller shaft (the red line indicates the deformed position of the upper roller shaft).

In order to determine the maximum stress and the maximum vertical displacement of the upper roller shaft using the Euler–Bernoulli theory, the principle of superposition was applied (see Figure 9) because the upper shaft behaves elastically under each individual load, namely: (i) the pre-tensioning force $F_{p}$ (loading stage I, Figure 9a), (ii) the vertical component of the rolling force (loading stage II, Figure 9b, in which q is the uniformly distributed rolling force), and (iii) the shaft weight that is active during the rolling process (loading stage III, Figure 9c, in which $\tilde{q}$ is the own weight of the upper roller shaft).

In practice, the pre-tensioning forces are limited to a maximum value, depending on the design configuration of the system (i.e., the diameter and the material of the upper roller shaft).

The vertical displacements in the plane of symmetry of the shaft can be written as follows:

(25) $$v_{S}^{I}=\frac{F_{p}\times l_{1}\times {\left(l_{2}+l_{3}\right)}^{2}}{2EI}\;for\;stage\;I$$

(26) $$v_{S}^{II}=\frac{q\times l_{3}}{24EI}\left(8\times l_{2}^{3}+5\times l_{3}^{3}+{24\times l}_{3}\times l_{2}^{2}+20\times l_{3}^{2}\times l_{2}\right)\;for\;stage\;II$$

(27) $$v_{S}^{III}=\frac{5\times \tilde{q}\times {\left(l_{2}+l_{3}\right)}^{4}}{24EI}-\frac{\tilde{q}\times l_{1}^{2}\times {\left(l_{2}+l_{3}\right)}^{2}}{4EI}\;\mathrm{for}\;\mathrm{stage}\;\mathrm{III}$$

in which $F_{p}$ (N) is the pre-tensioning force, *q* (N/m) is the uniformly distributed rolling force, $\tilde{q}$ (N/m) is the uniformly distributed load from the shaft weight, *E* is the Young’s modulus, and *I* is the moment of inertia of the cross-section.

The bending moment in the plane of symmetry of the shaft is given by:

(28) $$M^{I}=F_{p}\times l_{1}\;for\;stage\;I$$

(29) $$M^{II}=-\frac{q\times l_{3}^{2}}{2}+q\times l_{3}\times \left(l_{2}+l_{3}\right)\;for\;stage\;II$$

(30) $$M^{III}=\frac{\tilde{q}\times {\left(l_{2}+l_{3}\right)}^{2}}{2}-\frac{\tilde{q}\times l_{1}^{2}}{2}\;\mathrm{for}\;\mathrm{stage}\;\mathrm{III}$$

in which *q* (N/m) is the uniformly distributed rolling force from 2D FE simulation, and $\tilde{q}$ (N/m) is the uniformly distributed load from the weight of the shaft.

According to the reference system in Figure 9, the sign of the vertical displacement and bending moment is “-“ for loading stage I and III and “+” for loading stage II.

The total bending moment *M* in the central section of the upper roller shaft is obtained by summing the bending moments in all three loading stages (Equations (28)–(30)).

The maximum normal stress $\sigma_{max}$ is then calculated from the relationship

(31) $$\sigma_{max}=\frac{M}{W}$$

in which $W=\frac{\pi \times D_{wu}^{3}}{32}$ is the section modulus and $D_{wu}$ is the diameter of the upper roller shaft.

### 4.2. Calculation of the Maximum Stress, Displacement of the Upper Roller Shaft and Pre-Tensioning Forces Using FE Analysis

The calculation of the stresses of the upper roller shaft can be also carried out using parametric finite element modeling if an optimization module is available. In this study, the Ansys Workbench (version 19.0, 2019, Ansys, Inc., Canonsburg, PA, USA [23]) with the Static Structural module was coupled with the Response Surface module [23] that builds a continuous function of outputs versus inputs. The flow chart for calculating the maximum stresses and deformation of the upper roller shaft is given in Figure 8. In the optimization procedure, the pre-tensioning forces were parameterized as input variables. It was further assumed that the pre-tensioning forces applied at the end bearings of the upper roller shaft are identical.

The domain of variation for the pre-tensioning forces $D_{F_{P}}$ is defined as the following:

(32) $$D_{F_{P}}=\left(0,F_{P\;max\;allowed}\right)$$

where $F_{P\;max\;allowed}$ is the maximum value allowed for the pre-tensioning force.

For a given value of the pre-tensioning force $F_{P}$, one can calculate the following:

- The maximum equivalent stress on the upper roller shaft, $\sigma_{ech\;max}$;
- The maximum absolute vertical displacement in the work area (active zone) of the upper roller shaft, $v_{abs\;max}$.

In order to determine the optimum value of the pre-tensioning force, two optimization approaches can be considered, according to the goal and expected outcome (Figure 8).

If the lowest possible value of the maximum equivalent stress $\sigma_{ech\;max}$ is the desired output, the optimization problem can be formulated as follows:

(33) $$\underset{F_{P\in }D_{F_{P}}}{\min}\left\{\sigma_{ech\;max}|v_{abs\;max}\leq v_{allowed}\right\}$$

If the lowest possible value of the absolute maximum displacement $v_{abs\;max}$ is the desired output, then the optimization problem can be formulated as follows:

(34) $$\underset{F_{P\in }D_{F_{P}}}{\min}\left\{v_{abs\;max}|\sigma_{ech\;max}\leq \sigma_{allowed}\right\}$$

In the above relationships, $v_{allowed}$ is the allowed maximum displacement of the upper roller shaft and $\sigma_{allowed}$ is the allowed equivalent stress on the entire upper roller shaft. The maximum absolute displacement can be calculated starting from the maximum $v_{max}$ and minimum $v_{min}$ displacement using the following relationship:

(35) $$v_{abs\;max}=max\left(abs\left(v_{min}\right),abs\left(v_{max}\right)\right).$$

From a practical point of view, the first formulation (Equation (33)) is advantageous if the goal is to determine the maximum stress and increase the lifetime of the upper roller shaft, while the latter (Equation (34)) is preferable if the aim is to ensure product consistency and accuracy.

However, the two formulations can be combined as follows:

(36) $$\underset{F_{P\in }D_{F_{P}}}{\min}\left\{\alpha \cdot \sigma_{ech\;max}+\beta \cdot v_{abs\;max}|v_{abs\;max}\leq v_{allowed},\sigma_{ech\;max}\leq \sigma_{allowed}\right\}$$

where α and β are the weighting coefficients. Depending on the values of the two coefficients, various compromise solutions can be obtained.

## 5. Results and Discussion

### 5.1. FE Simulation Results

**Figure 10:**
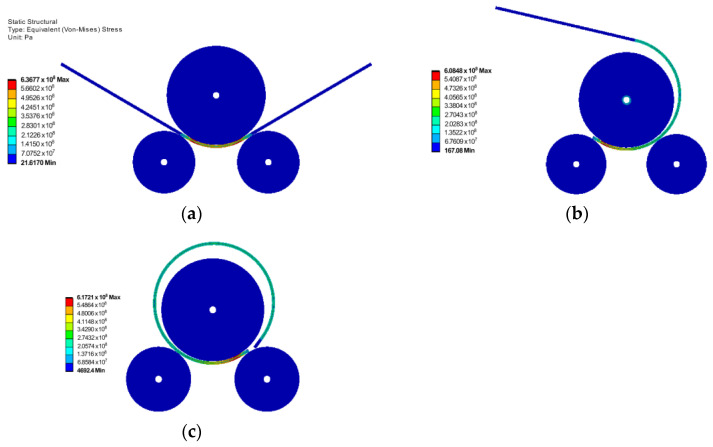
Von Mises stress (in Pa) at the end of the rolling stages for rolling an X70 plate with 23.6 mm thickness: (**a**) stage 1; (**b**) stage 2; (**c**) stage 3.

Figure 10 shows the distribution of the von Mises stress at the end of the three rolling stages, for rolling an X70 grade plate with a thickness of 23.6 mm into a pipe with the outer diameter of 864 mm. In the simulation, the upper roller has a diameter of 760 mm. It should be noted that, in this case, pre-deformation of the ends of the steel plate was not considered.

**Figure 11:**
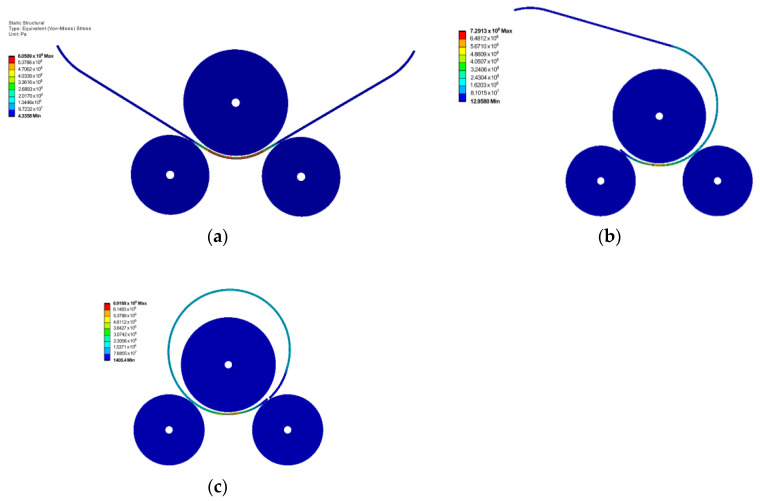
Von Mises stress (in Pa) at the end of the rolling stages for rolling an X70 plate with 20.6 mm thickness: (**a**) stage 1; (**b**) stage 2; (**c**) stage 3.

Figure 11 shows the distribution of the von Mises stress at the end of the three rolling stages for rolling an X70 plate with a thickness of 20.6 mm into a closed pipe using an upper roller shaft with a diameter of 640 mm. The corresponding outer diameter of the pipe was 813 mm. For this case, in order to successfully complete the simulation for all three stages and avoid any slipping of the rolled plate during the rolling process, the ends of the plate were pre-deformed, and this can be seen in Figure 11.

### 5.2. Rolling Force for Bending Angle Less or Equal to 41°

**Figure 12:**
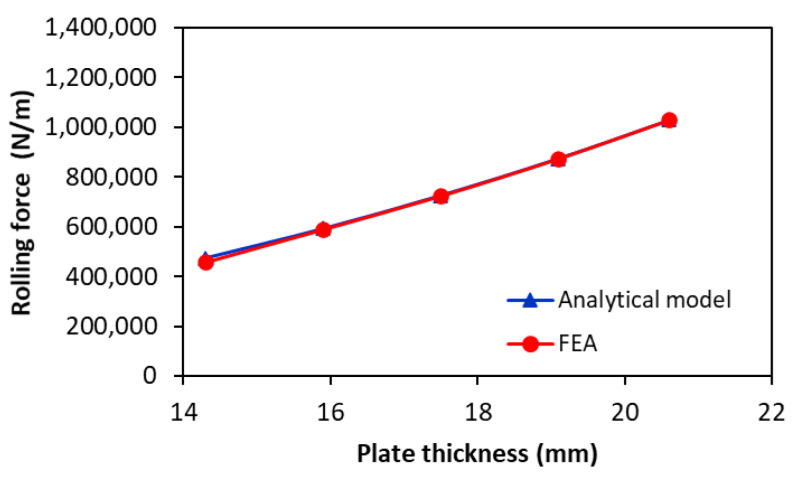
Analytical and numerically predicted rolling forces versus plate thickness for rolling X70 steel ($\alpha_{wl}=40.1{}^{\circ}$ and $K_{1}=1$).

Figure 12 presents the variation in the rolling force as a function of plate thickness for rolling X70 steel with a rolling system having an upper roller of 640 mm diameter. The results of the proposed analytical model (Equation (13)) are compared with those of the FE simulation to validate it under various conditions. As detailed in Section 3.1, the calculation of rolling force requires the selection of the $K_{1}$ reinforcement coefficient. Therefore, for this rolling system, the reinforcement coefficient is $K_{1}=1$ and $\alpha_{wl}=40.1{}^{\circ}$. It can be seen that the rolling force predicted by the proposed analytical model agrees very well with the FE calculation, with a maximum relative error of less than 4%. Figure 12 also indicates that the rolling force increases significantly with increasing plate thickness. With the increase in the plate thickness from 14 mm to 20 mm, the rolling force increases by about 225%.

**Figure 13:**
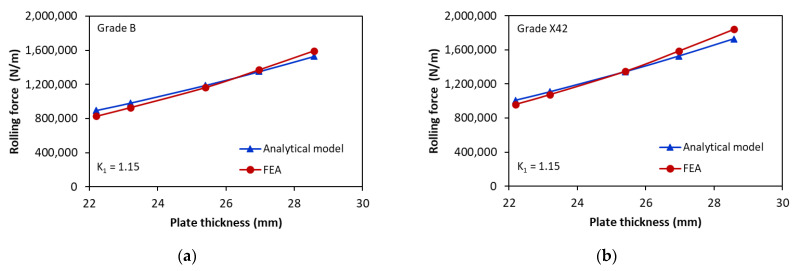

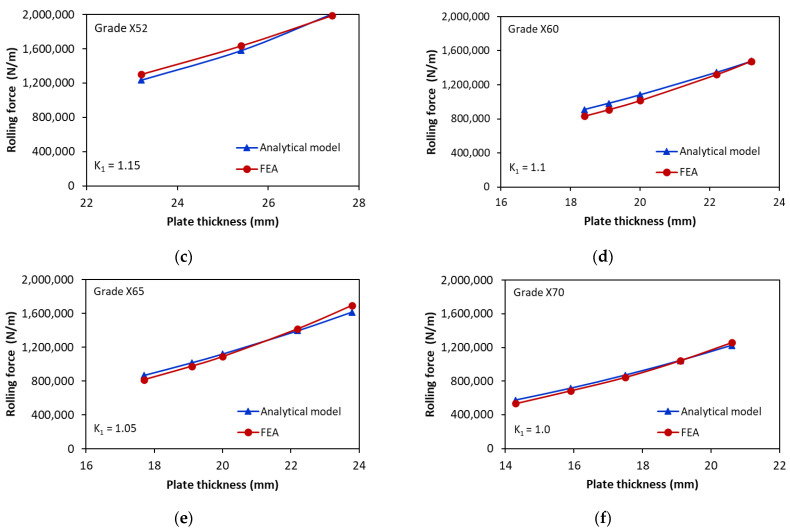
Rolling force versus plate thickness for bending angle $\alpha_{wl}=36.54{}^{\circ}$: (**a**) Grade B; (**b**) Grade X42; (**c**) Grade X52; (**d**) Grade X60; (**e**) Grade X65; (**f**) Grade X70.

In order to further validate the analytical model, Figure 13 compares the rolling force as a function of plate thickness, obtained using the analytical model and FE analysis, for rolling different steel grades (Grade B to Grade X70) with a wall thickness up to 28.58 mm. The rolling system consists of an upper roller of 760 mm diameter and two lower rollers of 480 mm diameter. This rolling system can be used to produce pipes with an outer diameter as large as 864 mm. It can be seen that both approaches agree very well for different plate thicknesses and steel grades. The maximum relative error of the rolling force predicted for these configurations with respect to the FE results is less than 8%. Therefore, the validity of the analytical model is confirmed, which confirms its use for predicting rolling force.

**Figure 14:**
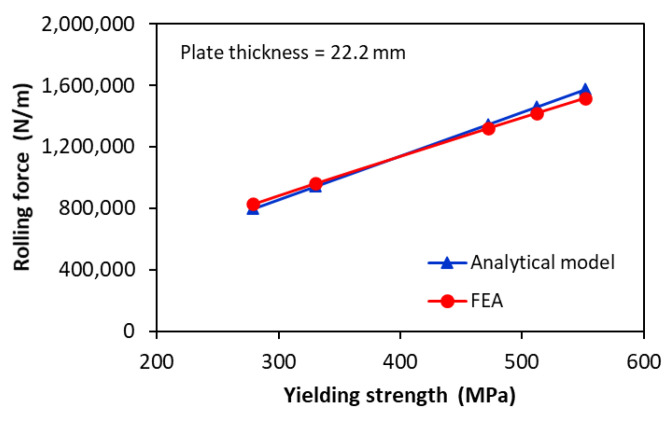
Rolling force versus yield strength for rolling a steel plate with 22.2 mm thickness ($\alpha_{wl}=36.54{}^{\circ}$, $K_{1}=1.1$).

The influence of the yield strength on the rolling force is illustrated in Figure 14 for rolling pipes with an outer diameter of 864 mm and 22.2 mm wall thickness using a three-roll bending system with a diameter of 760 mm for the upper roller and 480 mm for the lower rollers. The numerical simulation results are compared to analytical calculations obtained from Equation (13) where $\alpha_{wl}=36.54{}^{\circ}$, and $K_{1}=1.1$. Again, a very good agreement between the analytical and FE rolling forces was obtained, with respect to different yield strength, with a maximum relative error of about 4%.

### 5.3. Rolling Force for Bending Angle Greater than 41°

**Figure 15:**
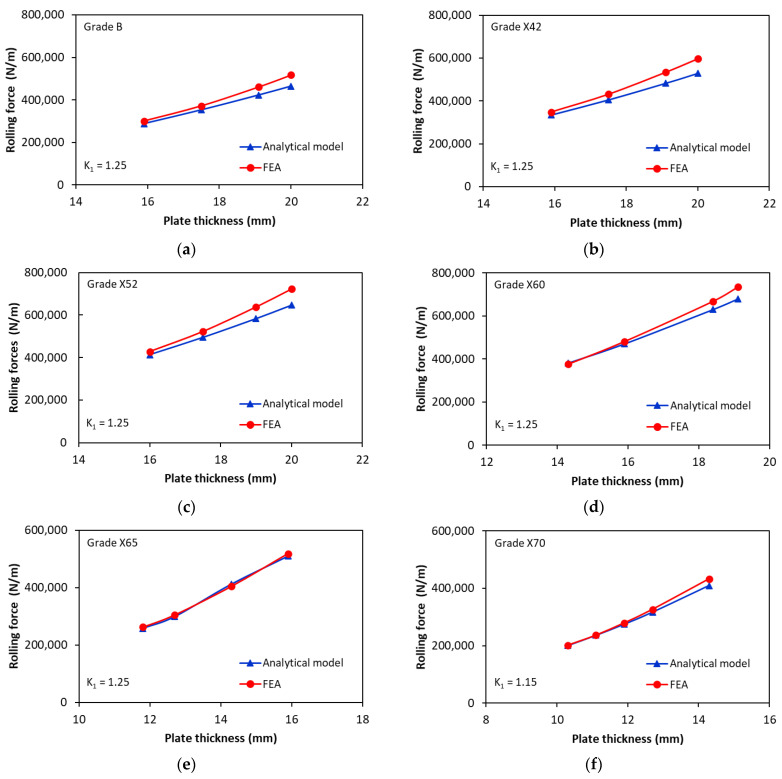
Comparison of analytical and numerical simulation results of the rolling force for a bending angle of $\alpha_{wl}=53.8{}^{\circ}$: (**a**) Grade B; (**b**) Grade X42; (**c**) Grade X52; (**d**) Grade X60; (**e**) Grade X65; (**f**) Grade X70.

Figure 15 shows the rolling force as a function of plate thickness for rolling different steel grades (from Grade B to Grade X70) with a roll bending system having an upper roller diameter of 420 mm and a lower roller diameter of 480 mm. This rolling system can be used to produce steel pipes with an outer diameter as large as 508 mm and a bending angle of 53.8°. In Figure 15, the rolling force values obtained with the analytical model (Equation (22)) are compared with the FE calculations.

Figure 15 indicates that the deviations between the FE and analytical rolling forces increased with increasing plate thickness and decreasing yield strength. Overall, the rolling force is realistically predicted by the analytical model, with a maximum relative error of less than 12%. However, for some grades, especially for those with higher yield strength, the analytical calculations for the rolling force are in very good agreement with the finite element calculations (maximum relative error of 8%, 6% and 5% for X60, X65, and X70, respectively), whereas the analytical model slightly underestimates the rolling force for grades with a lower yield strength (maximum relative error of 9%, 12% and 11% for Grade B, X42, and X52, respectively).

**Figure 16:**
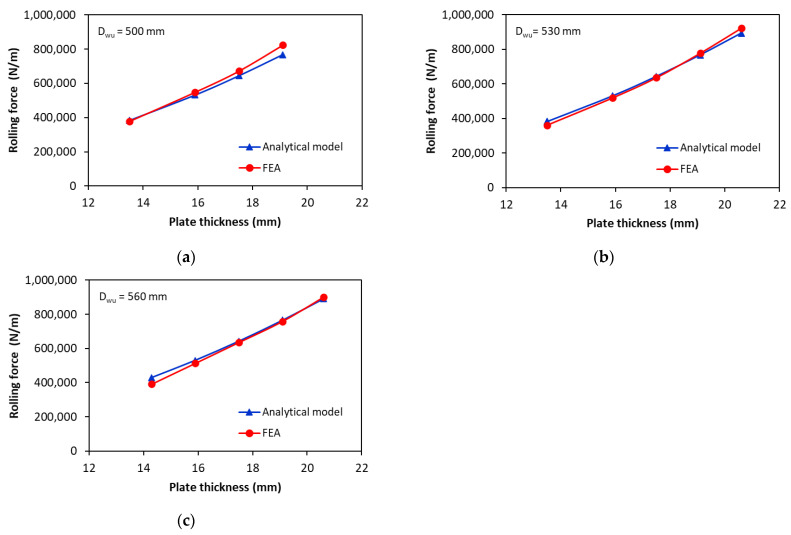
Effect of upper roller diameter on the rolling forces for the Grade X70: (**a**) $\alpha_{vi}=47.2{}^{\circ}$ and upper roller diameter of 500 mm; (**b**) $\alpha_{vi}=44.59{}^{\circ}$ and upper roller diameter of 530 mm; (**c**) $\alpha_{vi}=42.2{}^{\circ}$ and upper roller diameter of 560 mm.

Figure 16 compares the analytical and simulation rolling forces for X70 steel as a function of plate thickness for three different upper roller diameters (500 mm, 530 mm, and 560 mm). The results correspond to a reinforced coefficient $K_{1}=1.25$. It was observed that the rolling force obtained with the analytical model was in very good agreement with the FE calculation for the three upper rollers investigated, with a maximum relative error of less than 10% (7% for $D_{wu}$ = 500 mm, 6% for $D_{wu}$ = 530 mm, and 9% for $D_{wu}$ = 560 mm). Based on these findings, the validity of the model is also confirmed, which enables its use in the estimation of the rolling forces.

### 5.4. Calculation of the Pre-Tensioning Force, Maximum Stress and Displacement Using the Analytical Approach and FEA

**Figure 17:**
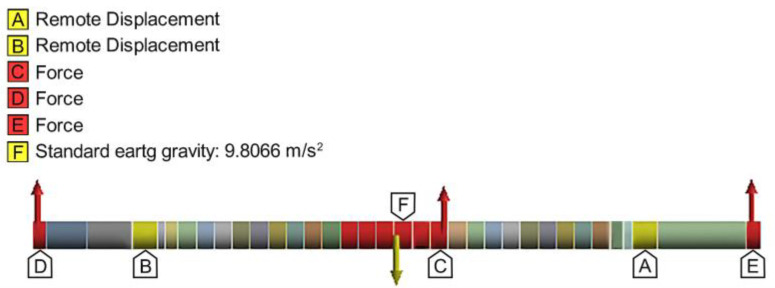
Boundary conditions for evaluating the state of stresses and deformations for the upper roller shaft.

To illustrate the applicability of the proposed methodology in Section 4, as an example, pipe rolling of an X70 sheet with 14.3 mm thickness was considered. The parameters of the rolling system are summarized in Table 4 along with the corresponding rolling forces computed using FE analysis (see Section 2). The discretization of the upper roller shaft was performed with 1221 higher order 3D solid elements (type SOLID186) with 20 nodes and three degrees of freedom per node [23]. Figure 17 shows the boundary conditions considered in the FE simulation. The upper roller shaft is made of steel intended mainly for roll bending machines with a minimum yield strength of 450 MPa, maximum allowable stress of 225 MPa, and density of 7850 kg/m^3^.

The results of the FE optimization procedure (i.e., pre-tensioning force, maximum absolute displacement, and maximum equivalent stress) for the case in which the value of the maximum stress is the desired output and the absolute maximum displacement is minimized are given in Table 5, for three points in the optimization procedure.

**Figure 18:**
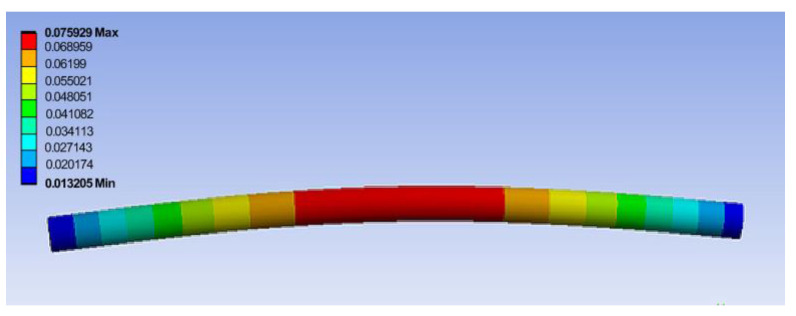
Distribution of vertical displacement (in m) with a maximum value in the central area of the upper roller shaft.

**Figure 19:**
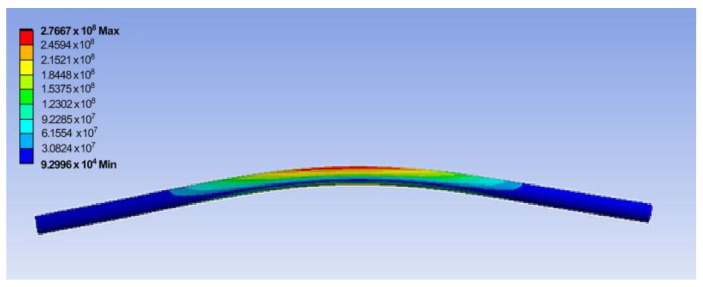
Distribution of the equivalent von Mises stress (in Pa) with a maximum value in the central area of the upper roller shaft.

Figure 18 shows the maximum displacement for the central area of the upper roller shaft that was parameterized as the results of the analysis, while the maximum von Mises stress that was parameterized on the entire surface of the shaft is presented Figure 19.

The results of the FE optimization procedure (i.e., the pre-tensioning force, maximum absolute displacement, and maximum equivalent stress) for the case in which the value of the absolute maximum displacement (imposed below the value of 10 mm) is important and the maximum stress is minimized are given in Table 6, for three candidate points.

**Figure 20:**
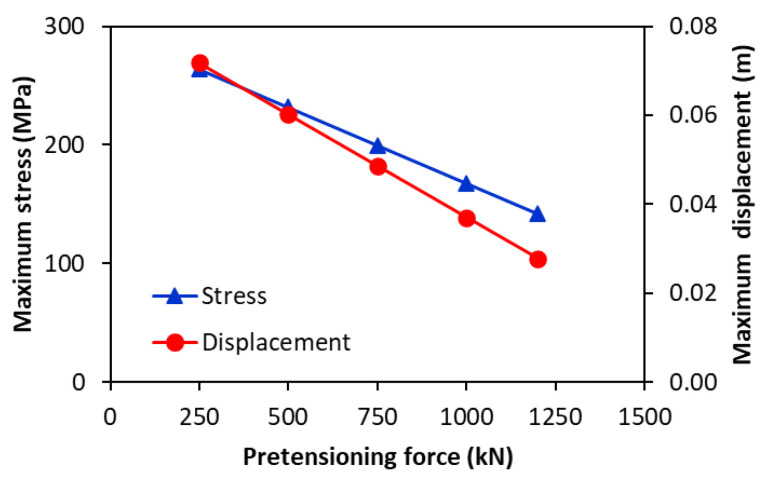
Variations in the maximum stress and vertical displacement in the central area of the shaft as a function of the pre-tensioning force based on the analytical approach.

The variations in the maximum stress (Equation (31)) and maximum vertical displacement (Equations (25)–(27)) in the central area of the upper shaft as a function of the pre-tensioning force using the analytical formulations are illustrated in Figure 20, for rolling X70 steel plate into a pipe with the outer diameter of 762 mm and 14.3 mm wall thickness. The diameter of the upper roller shaft was set to 620 mm, while the rolling length was set to 6 m. It is observed that the analytical formulation predicts a linear variation in the maximum stress and displacement with pre-tensioning forces (Figure 20).

In order to validate the analytical Euler–Bernoulli formulation (without shear), finite element simulation was performed with beam finite elements (Timoshenko theory) and SOLID186 finite elements [23]. The results from the analytical formulation for the maximum vertical displacement and maximum equivalent stress are compared with the numerical results of the FE simulations in Table 7.

It can be seen that the agreement between the analytical formulation and finite element simulation is very good, with an absolute relative error of 6% and 5% for the maximum displacement and equivalent stress, respectively. Therefore, it may be concluded that the maximum displacement and maximum equivalent stress can be adequately calculated using the analytical formulation based on the Euler–Bernoulli formulation.

## 6. Conclusions

In this paper, pipe rolling of steel plates using a three-roller bending system was investigated both analytically and numerically in order to determine the maximum rolling force. The three-roller bending process was modeled using a two-dimensional plane–strain model in ANSYS and the rolling force was determined as a function of plate thickness and upper roller diameter, for different types of API steel grades. Based on the numerical simulation results, the rolling systems were divided into two categories depending on the bending angle (e.g., less than or equal to, and greater than 41°), and two analytical models were developed for the prediction of the maximum rolling force. The analytical results for the rolling force agree well with the finite element results for different steel grades and plate thicknesses with maximum relative errors generally less than 10%.

The rolling forces were further used for the calculation of the stress and displacement of the upper roller shaft and the pre-tensioning forces necessary to obtain small vertical deformations in the central rolling area. To numerically determine an optimal value of the pre-tensioning force, two optimization approaches were proposed, by minimizing the maximum equivalent stress or the absolute maximum displacement. It was shown that for the upper roller shaft, the values of maximum stress and displacement obtained from the theoretical formulation based on the Euler–Bernoulli theory (without shear) were in good agreement with those obtained from the numerical simulation using finite elements of beam (Timoshenko theory) and solid finite elements.

Overall, the analytical models have the power to capture the complex mechanical response that occurs during the roll bending process of API steels, providing guidelines for such a difficult prediction problem.

Future work will address 3D modeling of the roll bending process with uneven distribution of rolling forces, taking into account the effect of pre-tensioning. In addition, in future work, design of experiment sensitive analysis will be implemented using the complete stress–strain behavior of the material systems.

## Figures and Tables

**Table 1:** Material properties used for finite element modeling [22].

Property	Rollers	Plate Steel					
	Steel	X70	X65	X60	X52	X42	B
Material behavior	Linear elastic	Bilinear elastic–plastic					
Young modulus (Pa)	2.1 × 10^11^	2.1 × 10^11^					
Tangent modulus (Pa)	-	2.1 × 10^9^					
Yield stress (MPa)	-	522	512	472	409	330	279
Poisson ratio	0.3	0.3					

**Table 2:** Characteristics of the rolling system.

Parameter (Unit)	Value
L (mm)	400
Dwl (mm)	480
Dwu (mm)	420, 500, 530, 560, 640, 760
t (mm)	14.3 to 28.58

**Table 3:** The reinforcement coefficient as a function of $t/R_{i}$ ratio.

${t/R}_{i}$	≤0.04	0.041–0.059	0.06–0.069	≥0.07
$K_{1}$	1	1.15	1.25	1.28

**Table 4:** Data of the rolling system.

Parameter	Value
*L*_1_ (m)	3
*L*_2_ (m)	3.86
*L*_3_ (m)	3
Rolling length (m)	6
Diameter of the upper roller (mm)	620
Outer diameter of the pipe (mm)	762
Thickness of the steel plate (mm)	14.3
q (kN/m)	457.6
$\tilde{q}$ (kN/m)	23.235

**Table 5:** Results from optimization based on minimization of the absolute vertical displacement.

Candidate Point	Pre-Tensioning Force (N)	Maximum Deformation (m)	Maximum Equivalent Stress $\sigma_{ech\;max}$ ≤ 225 × 10^6^ Pa	Maximum Absolute Displacement $v_{abs\;max}$ Minimization
1	1.725 × 10^6^	3.050 × 10^−3^	215.9 × 10^6^	3.050 × 10^−3^
2	1.725 × 10^6^	3.037 × 10^−3^	216.0 × 10^6^	3.060 × 10^−3^
3	1.726 × 10^6^	3.024 × 10^−3^	216.1 × 10^6^	3.069 × 10^−3^

**Table 6:** Results from optimization based on minimization of the maximum equivalent stress.

Candidate Point	Pre-Tensioning Force (N)	Maximum Deformation (m)	Maximum Equivalent Stress Minimization (Pa)	Maximum Absolute Displacement $v_{abs\;max}\;\leq \;0.01$ (m)
1	1552.325	9.987 × 10^−3^	191.957 × 10^6^	9.987 × 10^−3^
2	1552.855	9.964 × 10^−3^	192.021 × 10^6^	9.964 × 10^−3^
**3**	1553.298	9.945 × 10^−3^	192.074 × 10^6^	9.945 × 10^−3^

**Table 7:** Maximum vertical displacement and equivalent stress for the upper roller shaft: comparison between analytical and finite element values.

Approach	Max. Vertical Displacement (m)	Max. Equivalent Stress (Pa)
Analytical results—Euler–Bernoulli theory	0.07175	2.64 × 10^8^
Ansys beam—Timoshenko beam theory	0.07580	2.64 × 10^8^
FE Ansys—1221 SOLID186 elements	0.07590	2.77 × 10^8^
FE Ansys—160,379 SOLID186 elements	0.07582	2.77 × 10^8^

## Data Availability

The original contributions presented in the study are included in the article; further inquiries can be directed to the corresponding author.
